# A new species of *Nicon* Kinberg, 1866 (Polychaeta, Nereididae) from Ecuador, Eastern Pacific, with a key to all known species of the genus

**DOI:** 10.3897/zookeys.269.4003

**Published:** 2013-02-18

**Authors:** Jesús Angel de León-González, Berenice Trovant

**Affiliations:** 1 Universidad Autónoma de Nuevo León, Facultad de Ciencias Biológicas, Ap. Postal 5, Suc. “F”, San Nicolás de los Garza, Nuevo León, 66451, México; 2 Centro Nacional Patagónico (CONICET), Boulevard Brown 2915, U9120ACF Puerto Madryn, Chubut, Argentina

**Keywords:** Annelida, polychaetes, Nereididae, intertidal, Ecuador, taxonomy, systematics

## Abstract

A new species of *Nicon* Kinberg, 1866 from the east Pacific coast of Ecuador is described. The new species is characterized by a long, thin dorsal ligule on median and posterior parapodia and infracicular sesquigomph falcigers in the neuropodia. A key to all species of *Nicon* is provided.

## Introduction

Ecuador possesses a great variety of coastal environments resulting in a high diversity of marine species; however, taxonomic studies on marine invertebrates are few, especially in the case of the polychaetes. In Ecuador (excluding the Galapagos), only 29 families, 53 genera and 75 species of polychaetes have been recorded. Hartman (1939) was the first to report on the polychaetes from Ecuador and described four new species and ten new records from Puna and Santa Clara Islands (Guayas Province). Later, Cruz et al. (1980) provided four new records from benthic samples collected on the Estero Salado, Guayaquil Gulf. In the same Gulf, 29 species of polychaetes were identified by Villamar (1983). Villamar (1989) later reported marine species at Canal del Morro and Jambeli in the Guayaquil Gulf. Villamar and Cruz (2007) reported three taxa for Ecuador from the intertidal zone of Monteverde (Guayas Province). A new species of *Australonuphis*, used as fishing bait, was described by de León-González et al. (2008) in Santa Elena Bay (Guayas Province). In northern Ecuador very little is known about the polychaete fauna and only one ecological study has been carried out by Villamar (2006) in the intertidal zones of Manabi and Esmeraldas Provinces. In that paper he reported 27 species, of which 14 constituted new records for Ecuador. More recently, Trovant et al. (2012) reported 12 new species records in the Bunche and Cabo San Francisco intertidal sandy beaches of northern Ecuador (Esmeraldas Province).

The importance of the family Nereididae is manifested by their high diversity and abundance in all marine substrates, occurring in all oceans from the supralittoral to the abyssal zone. This family includes 44 genera and approximately 460 valid species (de León-González, 2009). *Nicon* is one of the least species rich genera of Nereididae. The genus was first described by Kinberg, (1866) for six species, *Nicon pictus*, *Nicon tahitianus*, *Nicon maculata*, *Nicon eugeniae*, *Nicon loxechini* and *Nicon virgini*, none of which were figured. In this paper, a new species of *Nicon* is described. It is characterized by having an elongate notopodial dorsal ligule, resembling a long cirrus on median and posterior parapodia, as well by the presence of sesquigomph falcigers in the neuropodia.

## Material and methods

**Figure 1.**
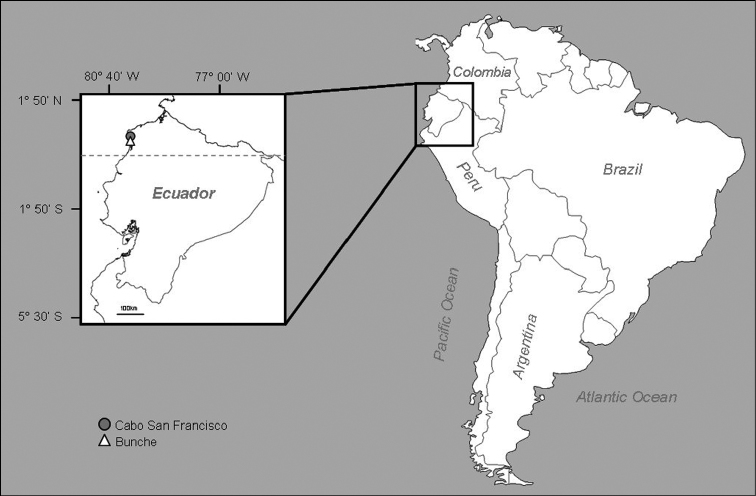
Map of Ecuador indicating the sampling sites, Bunche and Cabo San Francisco Beaches.

Samples were collected in March 2009 (dry season) in the intertidal zone of two sandy beaches located in the Esmeraldas Province, northern Ecuador (Fig. 1). Bunche beach (0°37'55"N, 80°02'14"W) is a protected area characterized as a low energy beach, with soft sloping banks and very fine particle sand, and Cabo San Francisco beach (0°39'11"N, 80°04'10"W) is characterized as a high energy environment, subjected to frequent and severe storms, with high slopes. Fresh-water discharges affect both beaches. Sediment samples were sieved through a 1mm mesh. Specimens were fixed in 10% formalin and later preserved in 70% ethanol. Terminology of parapodial structures was taken from Bakken and Wilson (2005). Type material has been deposited in the Natural History Museum of Los Angeles County, Allan Hancock Foundation Polychaete Collection (LACM-AHF), and the Polychaetological Collection of the Universidad Autónoma de Nuevo León (UANL).

## Results

### Systematics. Class Polychaeta Grube, 1850. Order Phyllodocida Örsted, 1843. Family Nereididae Lamarck, 1818

#### Nicon

Kinberg, 1866 emended

http://species-id.net/wiki/Nicon

##### Type species.

*Nicon maculata* Kinberg, 1866.

##### Diagnosis.

Prostomium pyriform to subpyriform, with two pairs of eyespots, paired frontal antennae and biarticulate palps. Four pairs of tentacular cirri with distinct cirrophores, smooth or articulated. Parapodia of first two chaetigers subbiramous, notopodium represented by a single ligule with dorsal cirri at its base. Subsequent notopodia with dorsal and ventral ligules with or without a small notopodial prechaetal lobe decreasing in far posterior parapodia. Neuropodia with superior and inferior prechaetal lobes, digitiform or conical postchaetal lobe present or absent along body, and a ventral ligule which can be reduced in posterior parapodia; ventral cirri short, tapered. All notochaetae homogomph spinigers; neurochaetae homogomph, heterogomph or sesquigomph falcigers, may be accompanied by homogomph and heterogomph spinigers, and simple chaetae. Pygidium with paired anal cirri. Pharynx with paired mandibles, without paragnaths or papillae.

##### Remarks.

This generic diagnosis was modified from Pettibone (1971), Wu and Sun (1979) and Hutchings and Reid (1990). Some important characteristics were not included by Pettibone (1971) because at that time she recognized *Nicon maculata* as the only member of the genus. Later on, Wu and Sun (1979) and Hutchings and Reid (1990) expanded the genus diagnosis including characters of recently described species such as *Nicon japonicus* Imajima, 1972, *Nicon yaguinae* Fauchald, 1972, *Nicon sinica* Wu & Sun, 1979 and *Nicon rotunda* Hutchings & Reid, 1990. Some new characters included in the present diagnosis are the presence-absence of a notopodial prechaetal lobe, and the occurrence of neuropodial sesquigomph falcigers.

#### Nicon orensanzi sp. n.

urn:lsid:zoobank.org:act:149CDBF9-ACD0-4D7E-8407-BD8ADF3955C6

http://species-id.net/wiki/Nicon_orensanzi

Figures 2 , 3

##### Material examined.

Holotype (LACM-AHF 4999), Paratype (LACM-AHF 5000) and Paratype (UANL 7840) collected at Bunche beach (0°39'01.98"N, 80°03'55.01"W), Esmeraldas Province, Ecuador, March 21 2009, coll. Berenice Trovant and Santiago Tineo. Additional material: seven anterior fragments, same data as holotype; two complete specimens and three anterior fragments, Cabo San Francisco beach (0°38'16.35"N, 80°3'14.07"W), Esmeraldas Province, Ecuador, March 20 2009, coll. Berenice Trovant and Santiago Tineo.

##### Description.

**Figure 2.**
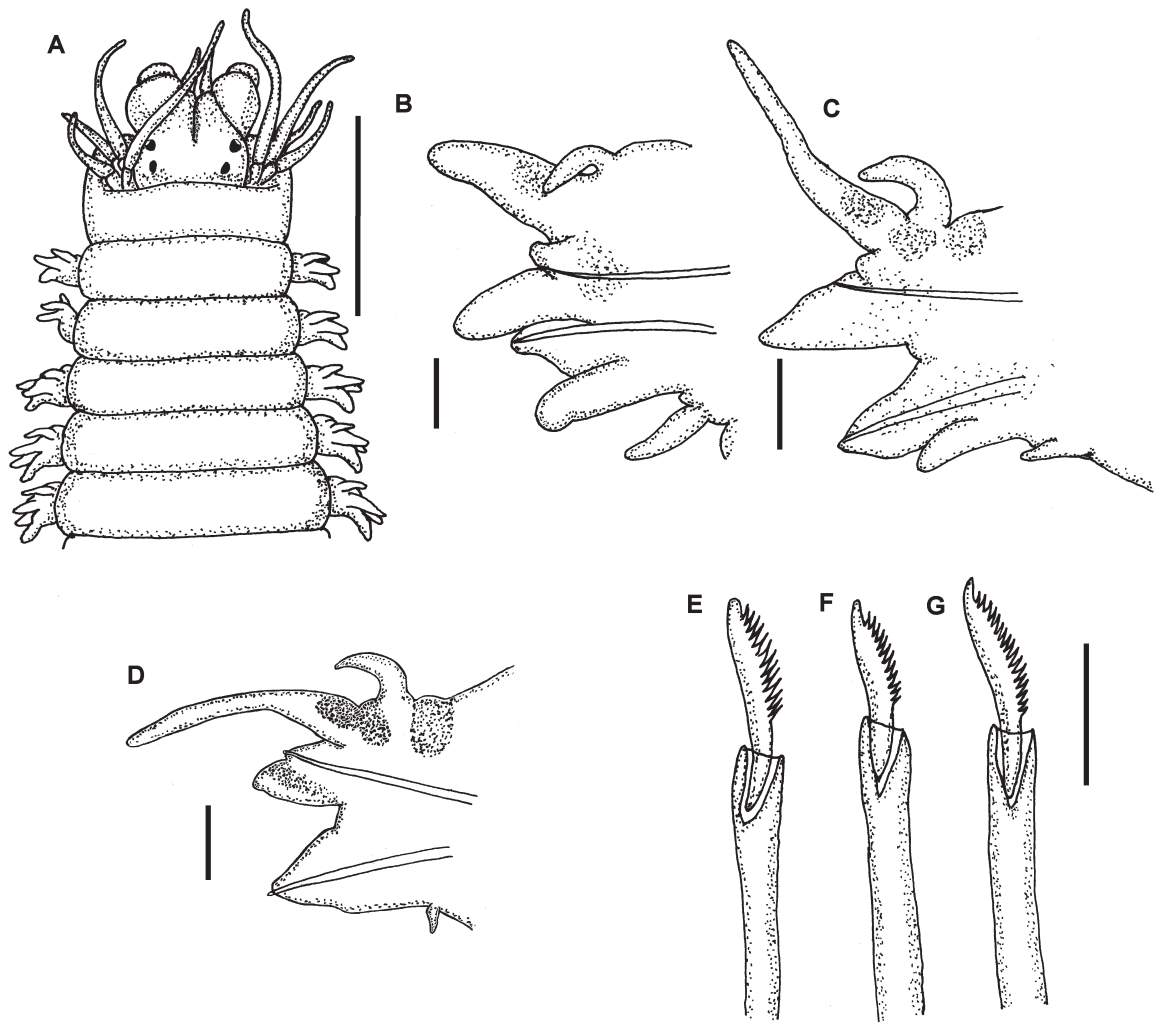
*Nicon orensanzi* sp. n. Holotype. **A** Anterior end, dorsal view **B** Parapodium 10, anterior view **C** Parapodium 25, anterior view **D** Parapodium 60, anterior view **E–G**. Infracicular sesquigomph falcigers of parapodia 10, 25 and 50 respectively. Scale bars: **A**= 1 mm; **B–D**= 100 µ; **E–G**= 30µ.

**Figure 3.**
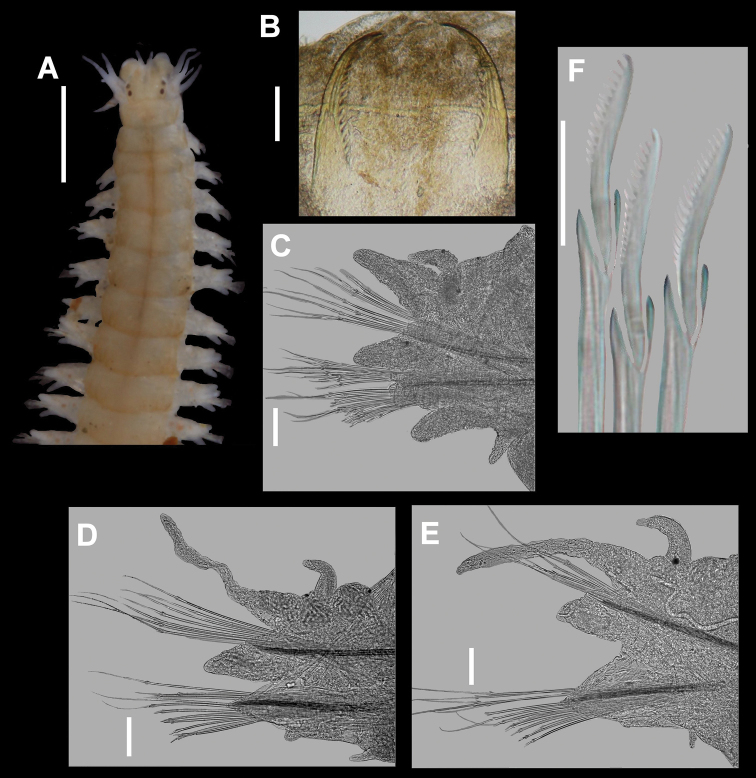
*Nicon orensanzi* sp. n. Paratype (UANL 7840). **A** Anterior end, dorsal view **B** Mandibles; Holotype (LACM) **C** Parapodium 9, anterior view **D** Parapodium 29, anterior view **E** Parapodium 62, anterior view **F** Infracicular sesquigomph falcigers of parapodium 62. Scale bars: **A**= 1 mm; **B**= 0.1 mm; **C–E**= 100 µ; **F**= 30µ.

Holotype incomplete posteriorly, with 85 chaetigers, 19mm long, 1.4mm wide. Prostomium pyriform, with frontal cleft extending to middle of prostomium. Two pairs of eyespots in trapezoidal arrangement, anterior pair slightly larger, with lenses. Pair of small cirriform antennae extending slightly beyond palps. Palps biarticulate, globose, with subspherical palpostyles. Peristomium longer than next segment, with four pairs of short tentacular cirri, longest reaching chaetiger two (Figs 2A, 3A). Pharynx lacking papillae or paragnaths, armed with pair of toothed mandibles (Fig. 3B). Anterior notopodia with short cirriform dorsal cirri, subtriangular dorsal ligule, and subulate notopodial ventral ligule. Small triangular prechaetal lobe, restricted to limited number of anterior chaetigers, reducing in size posteriorly, last present about chaetigers 28-30. Anterior neuropodia with superior and inferior lobe, subulate ventral ligule, ventral cirrus with inflated base (Figs 2B, 3C), postchaetal neuropodial lobe subulate, present in first 18 chaetigers, not visible in anterior view. Median and posterior notopodia with dorsal ligule long cirrus-like; prechaetal lobe absent, notopodial ventral ligule triangular, decreasing in size in posterior chaetigers. Median and posterior neuropodia with superior and inferior lobes poorly defined, neuropodial postchaetal lobe absent, neuropodial ventral ligule subulate, decreasing in size in posterior chaetigers until disappearing completely, ventral cirri cirriform, shorter than dorsal one (Figs 2C–D, 3D–E). All notochaetae homogomph spinigers, with long, thin blades. Anterior supracicular neurochaetae 6 long-bladed homogomph spinigers superiorly; 6 short-bladed heterogomph spinigers inferiorly. Anterior infracicular chaetae homogomph spinigers with long blade, and sesquigomph falcigers with anterior part ending in a blunt tooth (Fig. 2E). Median and posterior supracicular neurochaetae with long-bladed homogomph spinigers. Infracicular neurochaetae with a few homogomph spinigers superiorly, and sesquigomph falcigers inferiorly, anterior end sharper (Figs 2F–G, 3F). Pygidium lacking in holotype, with terminal anus and two thin lateral cirri on others specimens.

##### Type locality.

Bunche beach, Esmeraldas Province, Ecuador

##### Distribution.

This species is only known from Bunche and Cabo San Francisco beaches, Esmeraldas Province, Ecuador.

##### Discussion.

Of the six species originally included in the genus *Nicon* by Kinberg (1866) two have been transferred to other genera (*Nicon eugeniae*, currently *Nereis eugeniae* from Strait of Magellan and *Nicon loxochini*, currently *Platynereis magalhensis* from Strait of Magellan) and three species are considered indeterminable due to their incomplete descriptions and the poor condition of the available syntypes (*Nicon maculata* from La Plata, Argentina, *Nicon pictus* from Brazil, *Nicon tahitianus* from Tahiti, and *Nicon virgini* from Strait of Magellan) (Pettibone, 1971). Of these species, only *Nicon maculata* is considered valid at the present time. No type species was designated by Kinberg. Hartman (1949) designated *Nicon pictus* as the type species, even though she did not provide a diagnosis or figures. Pettibone (1971) later revised the genus and designated *Nicon maculata* as the type species. Currently this genus consists of ten species: *Nicon maculata* Kinberg, 1866 from La Plata, Argentina, *Nicon moniloceras* (Hartman, 1940) from Catalina Island, USA, *Nicon aestuarensis* Knox, 1951 from New Zealand, *Nicon polaris* Hartman, 1967 from the Antarctic peninsula, *Nicon abyssalis* Hartman, 1967 from the Antarctic peninsula, *Nicon japonicus* Imajima, 1972 from Japan, *Nicon yaquinae* Fauchald, 1977 from off the Oregon coast, USA, *Nicon sinica* Wu & Sun, 1979 from the Yellow Sea, *Nicon rotunda* Hutchings & Reid, 1990 from Australia, and *Nicon pettibonae* de León-González & Salazar-Vallejo, 2003 from the Loyalty Islands, New Caledonia. Pettibone (1971) also considered that *Nicon abyssalis* and *Nicon polaris* had doubtful generic affinities with *Nicon*; however, we believe that *Nicon abyssalis* possesses the generic characters of *Nicon* and therefore should be included in the genus. *Nicon polaris* was described based on an epitoke; however, the possession of an expanded elytra-shaped dorsal cirrus in the chaetiger 7 makes it doubtful that it belongs to *Nicon*; a similar structure is found in *Kainonereis*, currently a genus in *inquirenda* described from an epitokous stage by Chamberlin (1919).

Species of *Nicon* may be separated into two groups based on the presence or absence of notopodial prechaetal lobes. Those species with a notopodial prechaetal lobe are: *Nicon aestuarensis*, *Nicon japonicus*, *Nicon polaris*, *Nicon rotunda*, and *Nicon sinica*; while *Nicon abyssalis*, *Nicon maculata*, *Nicon moniloceras*, *Nicon pettibonae* and *Nicon yaquinae* lack a superior notopodial lobe. Some important characteristics of *Nicon* species are listed in Table 1.

*Nicon orensanzi* sp. n. is a member of the first group but differs in its long, thin notopodial dorsal ligule in median and posterior parapodia. *Nicon orensanzi* sp. n. and *Nicon pettibonae* are the only species in the genus with neuropodial infracicular sesquigomph falcigers in all parapodia. These two species differ in the shape of their sesquigomph falcigers, the presence of heterogomph falcigers, and a reduced dorsal ligule in the posterior parapodia of *Nicon pettibonae*.

**Etymology.** The new species is dedicated to Dr. José María (Lobo) Orensanz, who has made significant contributions to the taxonomy of polychaetes and has been a mentor to the authors of this paper.

**Table 1.** Diagnostic features of the species of *Nicon* (modified from Hutchings and Reid 1990). Abbreviations: TC= chaetiger number reached by longest tentacular cirri, ho sp= homogomph spinigers, he sp= heterogomph spinigers, ho f= homogomph falcigers, he f= heterogomph falciger, sf= sesquigomph falciger, DL= dorsal ligule, PL= Prechaetal lobe, ST= Subtriangular, SU= Subulate; DI= Digitate, CI= Cirriform, CO= Conical, E= Elongated.

**Species**	**Neuropodial chaetae**												
	**Supracicular**						**Infracicular**					**Notopodia**	
	**TC**	**ho sp**	**he sp**	**ho f**	**he f**	**sf**	**ho sp**	**he sp**	**ho f**	**he f**	**sf**	**DL**	**PL**
*Nicon abyssalis*	2	X	X	-	-	-	X	X	-	X	-	CI	-
*Nicon aestuarensis*	5	X	X	-	X	-	-	X	-	X	-	ST	X
*Nicon japonicus*	2	X	-	-	X	-	-	X	-	X	-	ST	X
*Nicon maculata*	10	X	-	-	X	-	X	-	-	X	-	SU	-
*Nicon moniloceras*	9	X	-	-	X	-	-	X	-	X	-	DI	-
*Nicon pettibonae*	5	X	-	-	X	X	X	-	-	X	X	ST	-
*Nicon polaris*	5	X	X	-	X	-	-	X	-	X	-	ST	X
*Nicon rotunda*	2	X	-	-	X	-	X	-	X	X	-	ST	X
*Nicon sinica*	9	X	-	-	X	-	-	X	-	X	-	CO	X
*Nicon yaguinae*	2	X	X	-	X	-	?	?	?	?	-	ST	-
*Nicon orensanzi* sp. n.	2	X	X	-	-	-	X	-	-	-	X	E	X

### Key to *Nicon* species

**Table:** 

1	Superior notopodial lobe present	2
–	Superior notopodial lobe absent	7
2	Tentacular cirri short, reaching chaetiger 2	3
–	Tentacular cirri reaching chaetiger 5	5
3	Heterogomph falcigers present on supra- and subacicular fascicle, dorsal ligule subtriangular	4
–	Heterogomph falcigers absent, with sesquigomph falcigers in infracicular position, dorsal ligule long and thin on median and posterior parapodia	*Nicon orensanzi* sp. n.
4	With homogomph falcigers in neuropodial subacicular position	*Nicon rotunda*
–	Homogomph falcigers lacking	*Nicon japonica*
5	Tentacular cirri reaching chaetiger 5, dorsal ligule subtriangular	6
–	Tentacular cirri reaching chaetiger 9, dorsal cirri conical	*Nicon sinica*
6	Mandibles with 6 oblique teeth, blade of falcigers short, with a terminal tooth directed downward	*Nicon polaris*
–	Mandibles with up to 10 teeth; blade of falcigers longer, with blunt terminal end	*Nicon aestuarensis*
7	Tentacular cirri short, reaching chaetiger 2	8
–	Tentacular cirri reaching chaetiger 5	9
8	Dorsal ligule cirriform , reduced in posterior chaetigers; falcigers with prolonged blade	*Nicon abysssalis*
–	Dorsal ligule subtriangular, similar in size throughout; falcigers with long, anteriorly blunt blade distinctly serrated along inner margin	*Nicon yaquinae*
9	Tentacular cirri reaching chaetiger 5; subtriangular dorsal ligule; supra and infracicular sesquigomph falcigers present	*Nicon pettibonae*
–	Tentacular cirri to chaetiger 9–10	10
10	Longest pair of tentacular cirri partially annulated on distal end; falcigers with long blade, denticulate along inner margin	*Nicon maculata*
–	All tentacular cirri annulated, with cylindrical articles; falcigers with short blades, denticles on proximal inner margin	*Nicon moniloceras*

## Supplementary Material

XML Treatment for Nicon

XML Treatment for Nicon orensanzi
